# Using patient-derived tumor organoids from common epithelial cancers to analyze personalized T-cell responses to neoantigens

**DOI:** 10.1007/s00262-023-03476-6

**Published:** 2023-06-27

**Authors:** Anup Y. Parikh, Robert Masi, Billel Gasmi, Ken-ichi Hanada, Maria Parkhurst, Jared Gartner, Sivasish Sindiri, Todd Prickett, Paul Robbins, Nikolaos Zacharakis, Mike Beshiri, Kathleen Kelly, Steven A. Rosenberg, James C. Yang

**Affiliations:** 1 Surgery Branch, National Cancer Institute, 10 Center Drive, Bldg 10 CRC 3W-5952, Bethesda, MD 20814 USA; 2 Department of Surgery, Morristown Medical Center, Morristown, NJ USA; 3 Laboratory of Genitourinary Cancer Pathogenesis, National Cancer Institute, Bethesda, MD USA

**Keywords:** Adoptive cell therapy, Immunotherapy, Organoids, Tumor-derived organoids, Immune evasion, HLA loss-of-heterozygosity

## Abstract

Adoptive cell transfer of tumor-infiltrating lymphocytes (TIL) can mediate durable complete responses in some patients with common epithelial cancers but does so infrequently. A better understanding of T-cell responses to neoantigens and tumor-related immune evasion mechanisms requires having the autologous tumor as a reagent. We investigated the ability of patient-derived tumor organoids (PDTO) to fulfill this need and evaluated their utility as a tool for selecting T-cells for adoptive cell therapy. PDTO established from metastases from patients with colorectal, breast, pancreatic, bile duct, esophageal, lung, and kidney cancers underwent whole exomic sequencing (WES), to define mutations. Organoids were then evaluated for recognition by autologous TIL or T-cells transduced with cloned T-cell receptors recognizing defined neoantigens. PDTO were also used to identify and clone TCRs from TIL targeting private neoantigens and define those tumor-specific targets. PDTO were successfully established in 38/47 attempts. 75% were available within 2 months, a timeframe compatible with screening TIL for clinical administration. These lines exhibited good genetic fidelity with their parental tumors, especially for mutations with higher clonality. Immunologic recognition assays demonstrated instances of HLA allelic loss not found by pan-HLA immunohistochemistry and in some cases WES of fresh tumor. PDTO could also be used to show differences between TCRs recognizing the same antigen and to find and clone TCRs recognizing private neoantigens. PDTO can detect tumor-specific defects blocking T-cell recognition and may have a role as a selection tool for TCRs and TIL used in adoptive cell therapy.

**Supplementary Information:**

The online version contains supplementary material available at 10.1007/s00262-023-03476-6.

## Introduction

In the evolving landscape of cancer treatment, the role of immunotherapy has rapidly increased to become a major component in the therapy of multiple cancers. Within the discipline of immunotherapy, the importance of cancer-specific mutated antigens (“neoantigens”) has also become clear. This has been shown for both immune checkpoint inhibition as well as for adoptive T-cell therapy. Retrospective studies on patients treated with both anti-CTLA4 and anti-PD1 antibodies showed an association between a higher mutational burden and improved survival [1]. Perhaps the best demonstration of this principle was the discovery that the high mutation rate associated with microsatellite instability seen in tumors with DNA repair defects was associated with a significantly higher response rate to checkpoint blockade than in patients whose tumors did not bear those defects, regardless of tumor histology [2].

One direct way to target neoantigens is through the use of adoptive cell therapy (ACT), which involves the transfer of in vitro expanded T-cells. This was initially done using tumor-infiltrating lymphocytes (TIL) from melanoma [3], which were subsequently shown to contain T-cells recognizing neoantigens [4]. This approach has been used with considerable success in patients with metastatic melanoma, inducing clinical objective response rates of 50–70%, including complete tumor regression rates of 20–25% [5, 6]. Adoptive transfer of neoantigen-reactive TIL has also been applied to patients with other malignancies, achieving durable complete responses in patients with cholangiocarcinoma [7], breast cancer [8], colon cancer [9], and cervical cancer [10].

In an effort to facilitate ACT for common epithelial cancers, protocols have begun using peripheral blood lymphocytes (PBL) genetically engineered to express cloned neoantigen-reactive T-cell receptors (TCRs) [11–13] to construct T-cell repertoires of the desired phenotype and specificity. These T-cell receptor engineered products target consistent, common hotspot mutations in KRAS [9, 14] and TP53 [15], as well as unmutated and overexpressed cancer-germline antigens like NY-ESO-1 [16, 17] and MAGE-A3 [18]. Nevertheless, the native immune response to human tumors is dominated by patient-specific, private neoantigens. In one study which precisely defined the neoantigens recognized by TIL from 75 patients with gastrointestinal cancers, only one *protein* (KRAS) was found to be a mutated neoantigen more than once—all others were unique and patient-specific [11]. This presents a daunting problem for screening patient T-cells for neoantigen recognition. Current methods are slow, expensive and labor-intensive [12]. Investigators have relied on either electroporating minigenes encoding a tumor’s neoantigens into antigen presenting cells (APC) or incubating APCs with synthetic mutated peptides to create tumor cell surrogates. However, the use of normal APCs in this role neglects intrinsic tumor-specific defects in antigen processing and presentation that may interfere with immune recognition. Availability of the patient’s autologous tumor line would be a major advantage. Conventional methods of culturing autologous tumor lines were largely unsuccessful and often required many months of effort. Patient-derived tumor organoids (PDTO) have begun to fill the void in personalized in vitro tumor modeling. PDTO are three-dimensional cancer cell lines that can be established from surgically resected tumors [19]. They are grown in a three-dimensional matrix enriched with tissue-specific media optimized to promote long-term proliferation of cancer cells. PDTO can be established from many tumor types, including colorectal [20, 21], breast [22], pancreatic [23], prostate [24], liver [25], ovarian [26], and lung cancers [27]. Organoids recapitulate histopathologic characteristics and genomic features of the source tumor—including copy number alterations, mutational load, and individual cancer gene mutations [22, 24–26]. This technology has been applied to pharmacologic screens, with a recent review of several studies demonstrating drug-sensitivity of PDTO in vitro were predictive of clinical responses of those patients [28]. Yet patient-derived tumor organoids (PDTO) have not been widely used for immunological purposes. Most of these efforts have also not utilized T-cells of precisely defined reactivity where recognition (and the failure to recognize) can be well documented and analyzed. We endeavored to determine whether PDTO could be used to identify and define tumor-related defects in immune recognition by neoantigen-specific T-cells with the aim of using them as a selection tool for T-cell transfer therapy.

## Materials and methods

### Collection of patient tissue and consent

All samples were derived from study participants who granted written, informed consent to be enrolled on a clinical protocol approved by the Institutional Review Board at the NCI (Bethesda, MD). The study protocol was registered under https://clinicaltrials.gov under NCT00068003.

Patients included in this study were those amenable for a low-risk metastasectomy with the intent of growing TIL that would potentially later be used for T-cell treatments under various Surgery Branch clinical protocols.

### Tissue processing and PDTO establishment

Establishment of PDTO was performed in accordance to previously published protocols [24]. In brief, fresh specimens obtained from operative resections of solid tumor metastases were enzymatically digested (Miltenyi Biotec, 130-095-929). Bloody tumors were treated with 1 mL ACK Lysing Buffer. Tumor cells were suspended in 80% Matrigel (Corning, 356238) and 20% histology-specific media adapted from previous publications [21–23, 27, 29, 30] (Supplementary Tables S1–S6). Tumor-cell suspensions were plated around the circumference of a single well of a 6-well tissue culture-treated plate or 10 cm tissue culture-treated dish and placed into a 37 °C CO_2_ incubator for 45 min. Once the Matrigel solidified, warmed histology-specific media was added to the center of the dish, which was placed back into a 37 °C CO_2_ incubator.

### Passaging of PDTO Cultures

Once cultures grew to confluency or large aggregates formed, PDTO lines were passaged, on average at 2–3 week intervals. Passaging of PDTO was also performed as per previously published protocols [24]. In brief, lines were treated with Dispase II (Gibco, 17105041) at a final concentration of 1 mg/ml for 2 h at 37 °C. Pelleted cells were subsequently treated with 1 ml TrypLE Express (Gibco, 12605-010) for 5 min with interval pipetting. Tumor cells were then suspended in Matrigel and media and plated as above.

### Next generation sequencing

Whole exome sequencing was performed on NextSeq550 models using SureSelectXT HS kit (Agilent, 5191-4029). RNA sequencing was on NextSeq550 models using Truseq RNA exome kits (Illumina, 20020189). Data processing and variant calling were performed as described previously [11]. Organoids were usually sequenced after 3–4 passages, which on average occurred about 8 weeks after initiation.

### Copy number analysis

Somatic copy number alterations and tumor purity estimates were determined using Sequenza R package [31] with a mutation frequency threshold adjusted to 0.08 from the default of 0.1 to account for intratumoral heterogeneity and normal tissue contamination.

### Clonality analysis

Cancer cell fractions (CCF) were determined using the variant allele frequencies from the exome data, and the tumor purity and copy number estimates obtained from Sequenza as inputs to Pyclone [32] run under default settings.

### HLA typing

HLA typing was determined by taking the consensus of two HLA-typing algorithms HLA-PRG-LA [33] & PHLAT [34].

### HLA loss of heterozygosity analysis

Class I HLA loss of heterozygosity analysis was performed using HLA-typing and cellularity and ploidy estimates of tumor with matched normal BAM files as input to an adjusted version of the original LOHHLA tool [35]. This custom version of the LOHHLA tool is deposited in Bitbucket: https://bitbucket.org/SENTISCI/lohhla/src/master/.

### qRT PCR analysis

RNA isolated from organoids and pancreatic cancer cell lines via the RNeasy Mini kit (Qiagen) was used to synthesize cDNA via reverse transcription. Custom-synthesized mutation-specific and mutation-unspecific primers and TaqMan Genotyping Master Mix were used to perform KRAS G12D-specific and total RAS (i.e., “Reference”) PCRs, which were run under thermocycling conditions of 95 °C for 10 min, followed by 50 cycles of 90 °C for 15 s and 60 °C for 1 min. Results were analyzed with the 7500 Fast Real-Time PCR System (Applied Biosystems) and presented relative to β-actin (ACTB) expression.

### Immunologic assays using PDTO

T-cells for testing were either bulk patient TIL or were produced by retroviral transduction of donor PBL with an α/β TCR of defined specificity and HLA restriction cloned from the TIL of a patient. The methodology of growing and identifying neoantigen-reactive TIL as well as the methodology of isolating and characterizing TCRs from neoantigen-reactive TIL cultures have been previously described [11]. In short, individual 4-1BB-positive T-cells from co-cultures of TIL and PDTO, mutant TMGs, or peptide-pulsed DCs were sorted by FACS and their TCRs were subsequently sequenced. Vectors encoding the TCRα and TCRβ chains were constructed and cloned into retroviral vectors, which were used to transduce autologous or allogeneic PBL and reactivity analyzed.

For co-culture assays, PDTO were passaged as described above and 24 h prior to assay, rested overnight in ultra-low adhesion 6-well plates (Corning, 3471) at 37 °C. They were then transferred at 1E5 cells per well in 100 μl of DMEM/10% FBS to a 96-well tissue culture-treated plate. Some PDTO were pulsed with synthetic minimal peptides at 1 μg/ml for 2 h at room temperature, washed three times before being added to the assay plate. Autologous or allogeneic T-lymphocytes retrovirally transduced with TCRs were added to the appropriate wells at a concentration of 5E4 to 1E5 cells per well in 150 μl. The human K562 erythroleukemia line and the primate COS-1 line were used to create positive and negative controls. Co-culture was performed for 18–20 h. ELISA for human IFN-γ secretion was performed on Day + 2 using the Human IFN-gamma DuoSet ELISA Development Kit (R&D Systems, DY285) as per the manufacturer’s protocol. Alternatively, IFN-γ ELISpot was performed on Day + 2. 96-well ELIIP plates (Millipore, MAIPSWU) were coated with IFN-γ capture antibody (Mabtech, clone: 1-D1K) overnight at 4 degrees F. Prior to co-culture, the plates were blocked with complete media for at least 1 h at room temperature (RT). Approximately 2e4 T lymphocytes were co-cultured with 0.5–1e5 dispersed organoid cells overnight at 37 °C in a humidified incubator. In some experiments, organoids were pretreated with IFN-γ prior to co-culture. After co-culture, cells were harvested from the ELISPOT plates into a standard 96-well round bottom plate for flow cytometry analysis; and then, the ELISPOT plates were washed 6 × with PBS + 0.05% Tween-20 (PBS-T), and then incubated for 2 h at RT with 100 μl/well of a 0.22 μm filtered 1 μg/ml biotinylated anti-human IFN-γ detection antibody solution (Mabtech, clone: 7-B6-1, diluent consisted of 1 × PBS supplemented with 0.5% FBS). The plate was then washed 3 × with PBS-T, followed by a 1 h incubation with 100 μl/well of streptavidin-ALP (Mabtech, diluted 1:3000 with above diluent). The plate was then washed 5 × with PBS followed by development with 100 μl/well of 0.45 μm filtered BCIP/NBT substrate solution (KPL, Inc.). The reaction was stopped by rinsing thoroughly with cold tap water.

## Results

### PDTO can be consistently and efficiently established from a variety of tumors

PDTO lines were initiated from 47 surgically resected fresh tumors from 36 patients (Supplementary Table S7). Successful establishment of an organoid line—defined by proliferation requiring one or more passages—occurred in 38/47 attempts. Of note, 17 additional organoid lines were established from patient-derived xenografts (PDX), though they were not included in this analysis. Success rates were similar across the major epithelial cancer types: 23/25 in colorectal carcinoma, 4/7 in breast carcinoma, and 4/5 in pancreatic carcinoma. For other histologies: 2/2 in cholangiocarcinoma, 1/1 in renal cell carcinoma, 1/1 in esophageal adenocarcinoma, 1/1 in anal squamous cell carcinoma, and 1/1 in non-small cell lung carcinoma. When grown specifically for the purposes of screening TIL for reactivity (*N* = 16), 75% of these PDTO attained 3E6 cells (the number required to screen TIL by co-culture) within 2 months, a timeframe typically needed to grow sufficient TIL to screen for reactivity.

Patient-matched TIL were screened for neoantigen recognition in nine patients who had PDTO established. The remainder were not screened against TIL due to clinical factors such as poor TIL growth or progressive disease rendering the patient ineligible for treatment. Some PDTO were also screened for recognition by reference T-cells (or TCR-transduced PBL) of allogeneic origin targeting specific shared neoantigens and HLA alleles on those organoids.

### PDTO are genetically faithful to the tumors from which they were derived

**Fig. 1:**
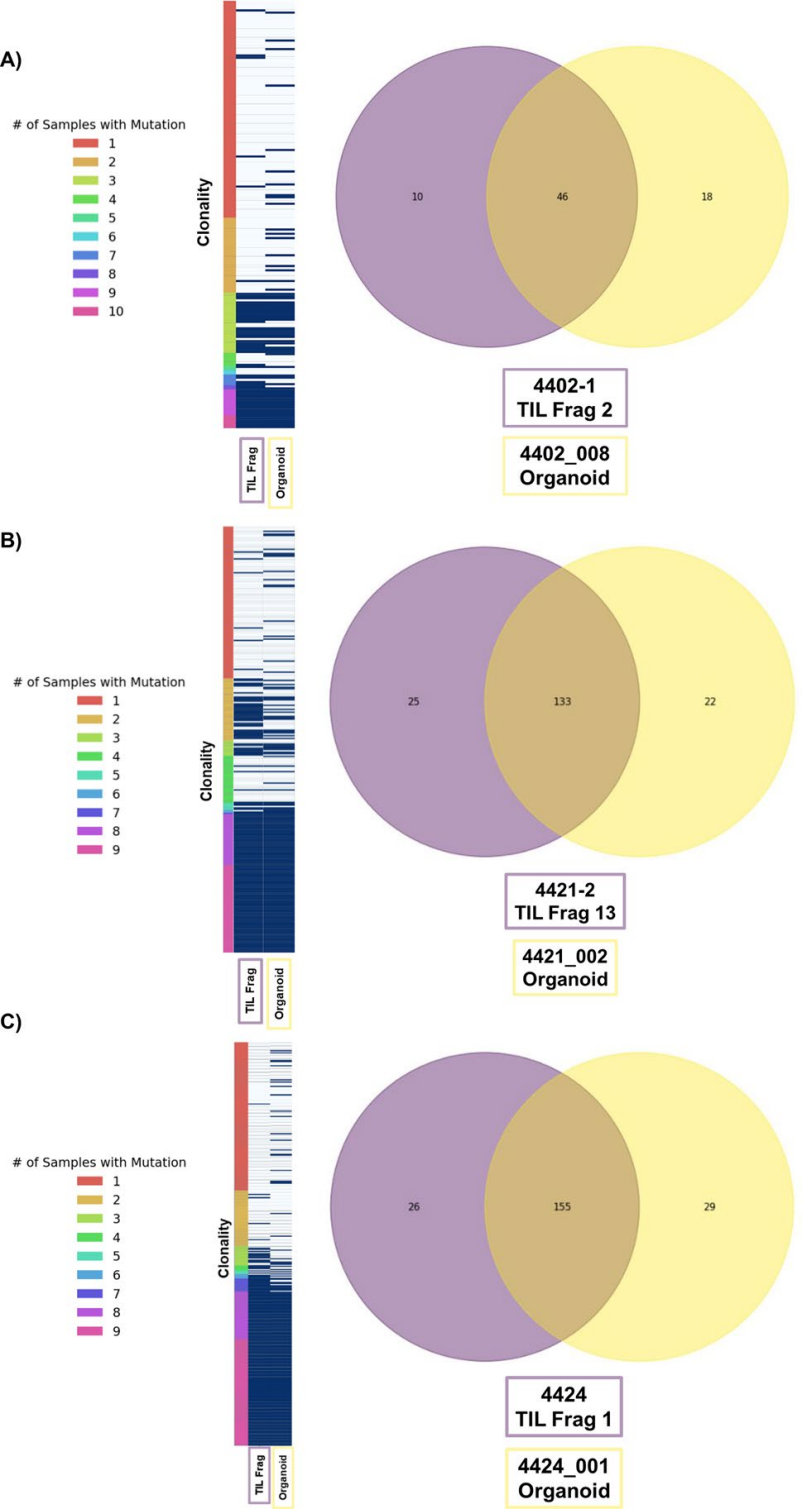
Patients had WES done on 6–11 independent tumor samples (multiple lesions and multiple samples within lesions). Each mutation found is represented as a horizontal line and ranked by its “clonality” on a color coded heatmap to the left (with mutations found once or twice in fresh tumor at the top and those found in all samples at the bottom). Its presence or absence in the organoid and its tumor fragment of origin are noted in the next two columns. Venn diagrams (on right) comparing overall tumor mutational burden in the organoid and original tumor fragment, shown for Patients 4402 (**a**), 4421 (**b**), and 4424 (**c**). Mutations with highest clonality are shared between the PDTO and original tumor fragment, with differences being within the least clonal of mutations

Whole exome sequencing (WES) was performed on an initial cohort of well-established PDTO in parallel with their parental fresh tumors. In the case of fresh tumors, WES was performed on different lesions, if available, as well as on multiple areas from the same lesion. These multiple sequences from the fresh tumors (6–11 per patient) were then compared to the WES of the PDTO to determine the fidelity of the mutational profile of PTDO (Fig. 1; Suppl Fig. S1. The most clonal somatic mutations (determined by their consistency across multiple sequences from fresh tumor) were consistently found in the derived PDTO, and often included known driver mutations. Genetic discrepancies between PDTO and fresh tumor were found mostly in subclonal mutations that may either be the result of mutational drift during PDTO growth or reflect true heterogeneity of these subclonal variants in the fresh tumor. Furthermore, the TMB was similar between these samples, with most of the total mutations shared between the PDTO and its parental tumor. PDTO 4433 (Suppl Fig. S1) initially had fewer mutations identified than its tumor fragment of origin. At that time of that sequencing, the early passage PDTO still contained significant normal tissue (by cytology). By passage nine, the tumor fraction was approximately 100% and genetic fidelity was much higher (Suppl Fig. S1). PDTOs 4430 and 4437 had more mutations identified than their tumor fragments of origin, and these tumor fragments had low tumor cell fractions and depths of sequencing, illustrating how PDTO sequencing may help correct such deficiencies in sequencing fresh tumor. Nevertheless, for highly clonal variants (the variants of most interest), fidelity was very high.

### Verification of neoantigen recognition using autologous PDTO

**Fig. 2:**
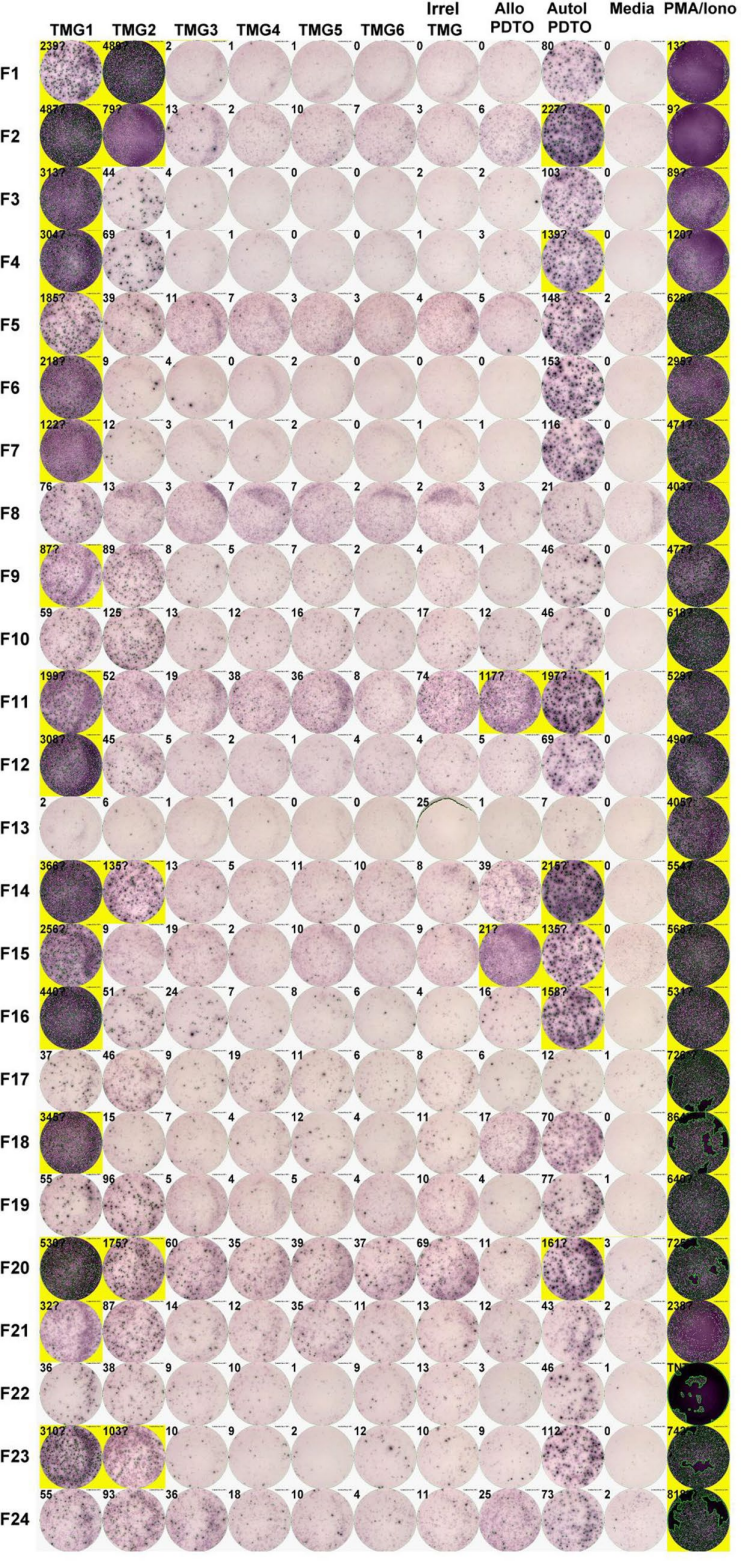
Correlation of TIL recognition of neoantigens expressed by tandem minigenes and TIL recognition of the autologous patient-derived tumor organoid. TIL fragment cultures labeled at left are tested against autologous dendritic cells electroporated with tandem minigenes TMG1-6 expressing tumor associated mutations, an irrelevant TMG control or against allogeneic (control) and autologous PDTO target cells. PMA stimulation of TIL cultures represent positive controls. Machine enumerated spot numbers are next to each spot. Highlighted wells were selected for further study

Our standard method of identifying TIL having neoantigen reactivity utilizes normal autologous dendritic cells to present tumor-specific mutations in the form of either tandem minigenes or synthetic peptides. Using normal dendritic cells negates the effects of tumor-specific defects in antigen processing or presentation that may affect the ‘real-world’ ability of these TIL to react with the autologous tumor. In addition, screening based on mutations found by WES ignores all classes of non-mutated tumor-associated antigens. The addition of PDTO to the screening assay may capture TIL reactive with mutated as well as non-mutated tumor antigens and may uncover defects in antigen processing and presentation that obviate the clinical utility of some antigenic targets. As an illustration, multiple TIL cultures derived from 24 separate fragments of tumor from a patient with colorectal cancer were tested against all non-synonymous mutations found by WES, encoded by tandem minigenes. Multiple TIL fragment cultures recognized mutated epitopes encoded in TMG1 and TMG2 (Fig. 2). To varying degrees, TMG-reactive TIL fragment cultures also recognized the PDTO and cultures with no recognition of TMGs also did not recognize the PDTO. Subsequently, TCRs specifically recognizing the neoepitopes KRAS G12D, GPATCH8 S784L and TRAPPC9 K588T were cloned from these TIL fragments (as described below). The first two neoantigens were encoded in TMG1 and TRAPPC9 K588T in TMG2. With multiple TIL cultures and multiple candidate neoantigens, patients can also show discordant results between peptide or TMG reactivity and PDTO reactivity. These discordant results can be the result of TIL recognizing non-mutated antigens (PDTO positive, DC screen negative) or may imply tumor-specific defects in antigen processing and presentation (DC screen positive, PDTO negative) and can be the basis of further investigation and personalization of adoptive T-cell therapy.

### PDTO can identify HLA loss in tumor causing a failure of T-cell recognition

**Fig. 3:**
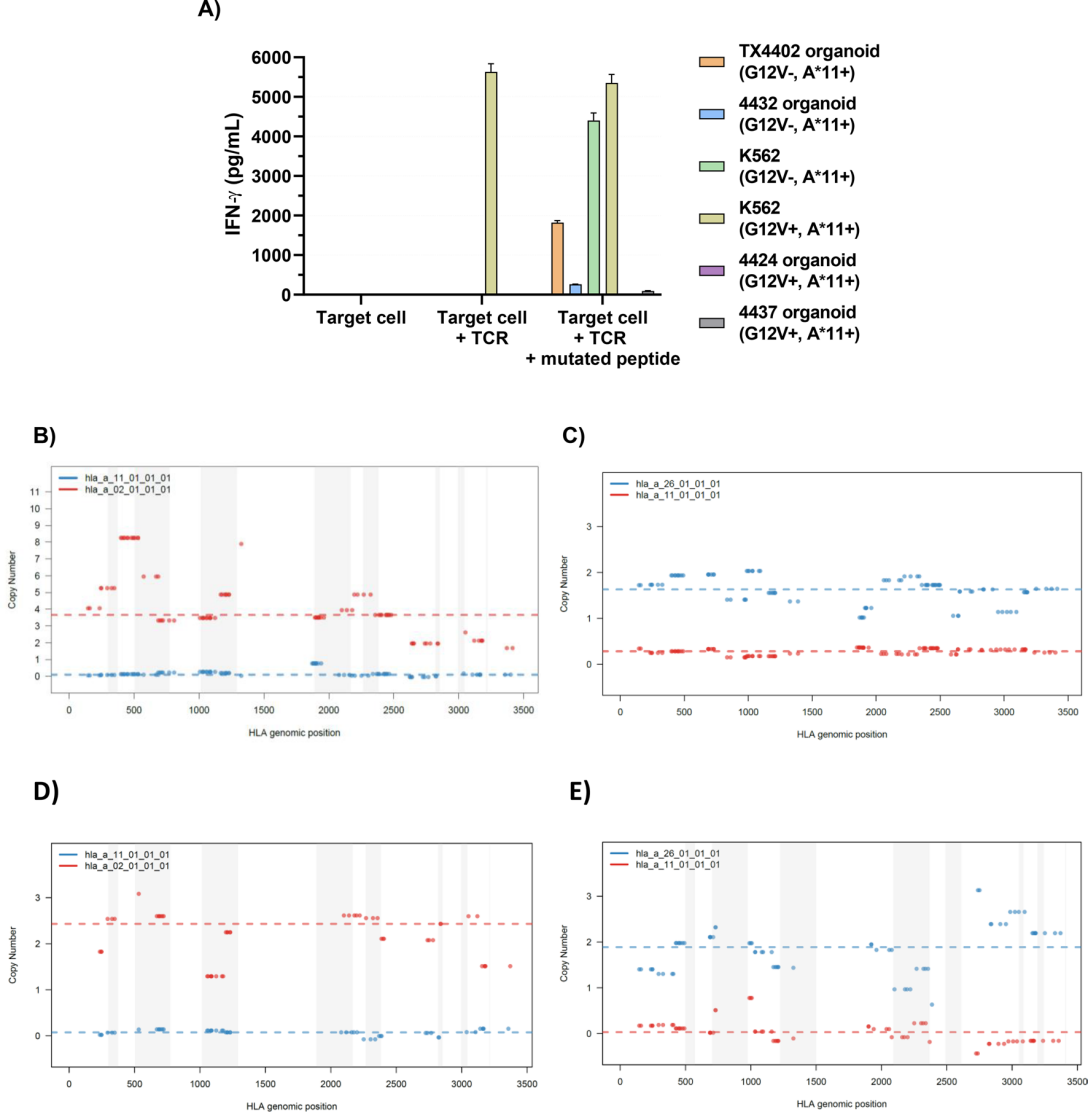
**a** A murine TCR specific for KRAS G12V and restricted by A*11:01 (raised in an HLA-A11 transgenic mouse) was retrovirally transduced onto A*11:01-negative donor T cells and co-cultured with the HLA-A11 + /G12V + 4424 and 4437 organoids. The G12V- TX4402 organoid and K562-A11 with and without G12V introduced were included as negative and positive controls. The assay was also conducted in the presence of added mutated peptide to provide exogenous antigen to test for HLA-A11 function. IFN-γ secretion was measured by ELISA. **b** HLA-LOH analysis via LOHHLA demonstrating loss of HLA-A*11:01 in 4424 organoid. **c** HLA-LOH analysis via LOHHLA demonstrating decreased HLA-A*11:01 in 4437 organoid. **d** HLA-LOH analysis via LOHHLA of fresh tumor sample from 4424. **e** HLA-LOH analysis via LOHHLA of fresh tumor sample from 4437

A common tumor-specific defect in antigen presentation that can be revealed by PDTO is simply the loss of the HLA presenting allele for a specific epitope. This would not be detected if autologous dendritic cells are used to present a patient’s neoantigen repertoire in a screening assay. PDTO lines were derived from a colorectal lung metastasis (4424) and a pancreatic soft tissue metastasis (4437) from HLA-A*11:01-positive patients with known KRAS G12V mutations confirmed to be retained in the PDTO. Our group previously isolated a murine TCR specific for KRAS G12V restricted by HLA-A*11:01 by vaccinating an HLA-A*11:01 transgenic mouse and showed this performs well in vitro and in vivo when introduced into human PBL [14]. PBL from an HLA-A*11:01-negative healthy donor (to avoid peptide presentation by the donor PBL) retrovirally transduced to express this TCR were co-cultured with the two PDTO and recognition was determined by IFN-γ release (Fig. 3a). There was no recognition of any of the G12V-negative control PDTO and the K562 transduced positive control was well recognized, yet there was no recognition of either of the 4424 or 4437 organoids. This was not rectified by pulsing the organoids with mutated peptide, suggesting HLA loss as a mechanism of immune evasion in these lines. LOHHLA analysis confirmed these findings (Fig. 3b–c), demonstrating decreased or total loss of HLA-A*11:01 in both organoids. Sequence from fresh tumor from these patients had low purities by Sequenza and although LOH was suggested for 4424, no definitive conclusion about LOH could be made for 4437 (Fig. 3d–e). The use of PDTO not only yielded clear sequence data from a source with high tumor purity, but the findings could be functionally verified by T-cell reactivity assays.

### PDTO can reveal poor immune recognition even when the HLA allele and the neoantigen are present

**Fig. 4:**
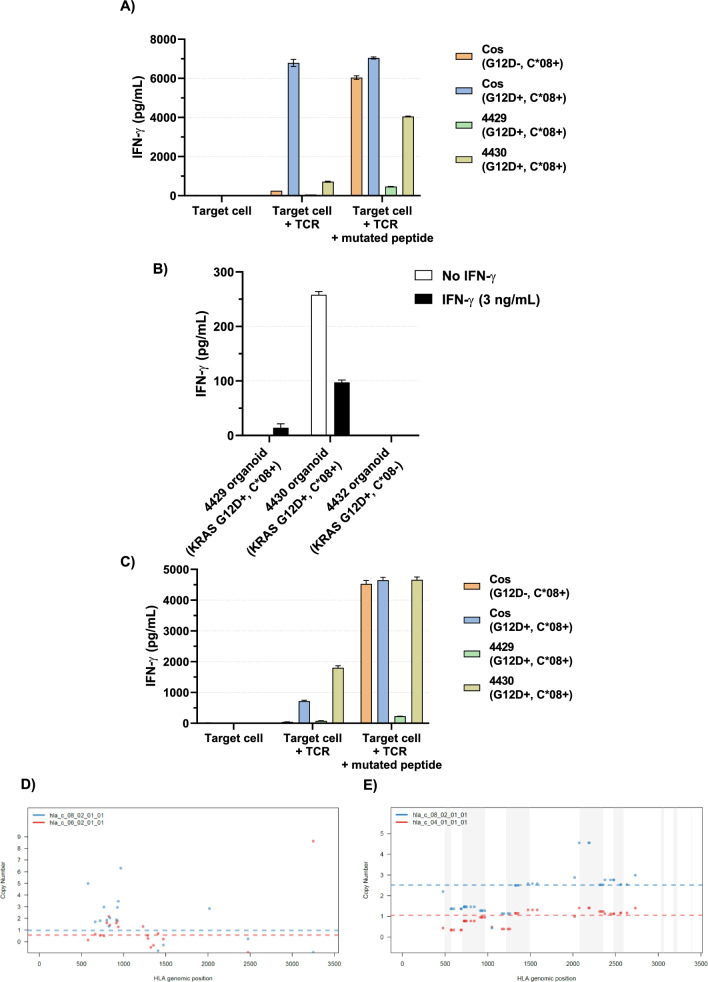
A patient-derived KRAS G12D-specific, C*08:02-restricted TCR recognizing the 9mer peptide (TCR #1) was retrovirally transduced onto C*08:02-negative donor T cells and co-cultured with 4429 and 4430 organoids, as well as with other C*08:02-expressing tumor cell lines with or without the KRAS G12D mutation (**a**). IFN-γ secretion was measured by ELISA. There was minimal recognition of either organoid unless the mutated peptide was pulsed onto its surface. IFN-gamma pretreatment did not significantly improve recognition of either organoid by TCR #1 (**b**). A different KRAS G12D C*08:02-restricted TCR recognizing the 10mer peptide (TCR #2) was retrovirally transduced onto C*08:02-negative donor T cells and co-cultured with 4429 and 4430 organoids (**c**). IFN-γ secretion was measured by ELISA. There was no recognition of the 4429 organoid unless the mutated peptide was pulsed onto its surface; the 4430 organoid was readily recognized without additional peptide. HLA-LOH analyses via LOHHLA demonstrated that HLA-C*08:02 was genetically present in the 4429 (**d**) and 4430 (**e**) organoid

PDTO lines were derived from colorectal metastases to lung (4429 and 4430) from HLA-C*08:02-positive patients with tumors known to have KRAS G12D mutations. The presence of the KRAS G12D mutation was confirmed by sequencing in the PDTO. Our group had previously isolated two TCRs that recognized the 9mer GADGVGKSA (TCR #1) and 10mer GADGVGKSAL (TCR #2) from KRAS G12D, presented in the context of HLA-C*08:02 [9]. The PDTO were co-cultured with HLA-C*08:02-negative healthy donor PBL retrovirally transduced to express either of these two TCRs and IFN-γ secretion measured. There was minimal recognition of either organoid by TCR #1 (Fig. 4a), however this could be enhanced by pulsing the 4430 organoid with the mutated 9mer epitope. This suggested the presence of functional HLA-C*08:02 on this PDTO. Poor recognition of the 4429 PDTO when pulsed with peptide indicated low expression of HLA-C*08:02 as a major component of its poor recognition by this TCR. When both PDTO were pretreated with IFN-γ to try to augment MHC class I expression and antigen processing, there was minimal effect on 4429 and a paradoxical decrease in recognition of 4430 (Fig. 4b). When tested against PBL transduced with TCR #2 (Fig. 4c), the 4429 organoid was again poorly recognized with minimal improvement after addition of exogenous peptide, while the 4430 organoid was readily recognized even without pulsing of mutant peptide. LOHHLA analysis confirmed HLA-C*08:02 was genetically present in both organoids (Fig. 4d–e). Taken together, these data suggest that 4429 has impaired HLA-C*08:02 expression unrelated to LOH and not responsive to IFN-γ; 4430, however, is recognized and those data also suggest that a T-cell expressing TCR #2 would be a better choice for therapy.

**Fig. 5:**
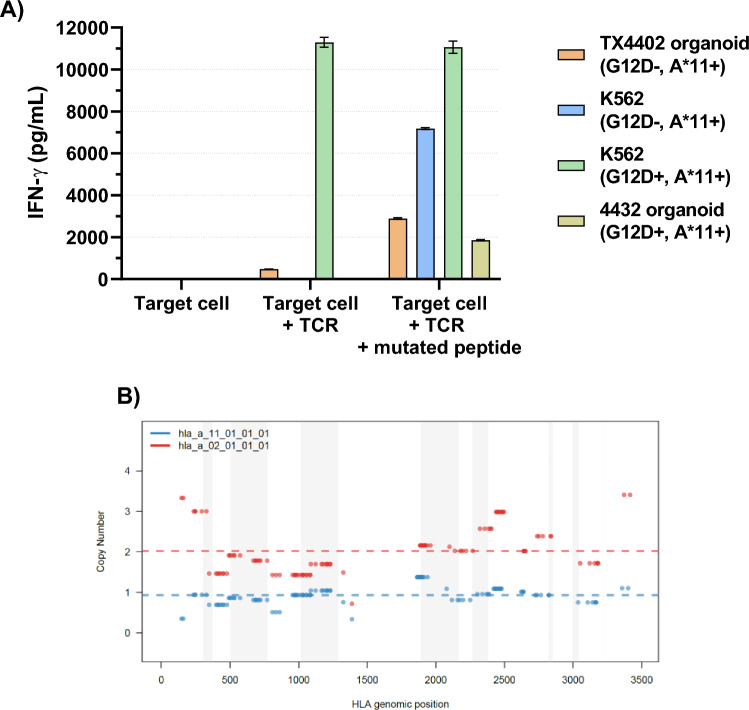
**a** A KRAS G12D-specific, HLA-A*11:01-restricted murine TCR (from an HLA-A11 transgenic mouse) was retrovirally transduced onto A*11:01-negative donor T cells and co-cultured with HLA-A11 + organoids, one with the G12D mutation (4432) and one without (TX4402, a breast cancer organoid stably transduced with HLA-A11), as well as with HLA-A11 transduced K562 with and without co-transduction of KRAS G12D. IFN-γ secretion was measured by ELISA. There was no recognition of the 4432 organoid unless the mutated peptide was pulsed onto its surface. **b** HLA-LOH analysis via LOHHLA demonstrated that HLA-A*11:01 was genetically present in the 4432 organoid

A PDTO line was additionally derived from another colorectal lung metastasis (4432) from an HLA-A*11:01-positive patient with a known KRAS G12D mutation retained in the PDTO. Using a TCR proven to recognize this peptide-MHC complex [14], this PDTO was co-cultured with HLA-A*11:01-negative healthy donor PBL retrovirally transduced to express this TCR and recognition determined (Fig. 5a). There was no recognition of this organoid by this TCR until mutated peptide was exogenously added. With LOHHLA analysis demonstrating intact HLA-A*11:01 in this organoid (Fig. 5b), this again suggested decreased MHC-peptide complex on the tumor surface due to insufficient expression of the KRAS G12D allele or a defect in its processing and loading onto MHC.

**Fig. 6:**
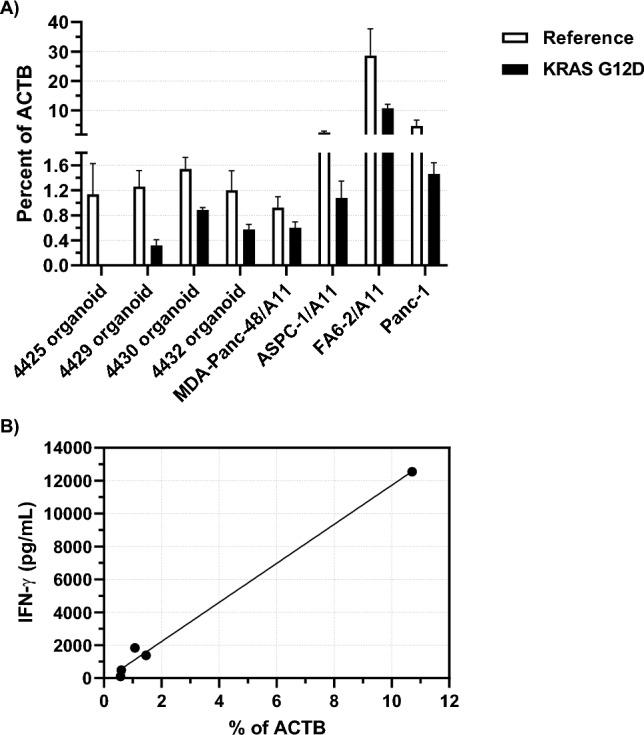
**a** qRT PCR was used to compare expression levels of mutated KRAS G12D RNA (compared to actin B) for a variety of organoids and conventional pancreatic tumor lines irrespective of HLA type (PDTO 4425 is a wild type KRAS control). **b** For the five tumors in **a** which expressed HLA-A11 (PTDO 4432, Panc-1 and the three tumor lines transduced with A11), IFN-g release when co-cultured with PBL engineered to express the KRAS G12D-specific A11-restricted TCR correlated with KRAS G12D expression (*R*^2^=0.9916, *p* < 0.0001)

Quantitative RT-PCR analysis demonstrated lower KRAS G12D mRNA expression in these three PDTO compared to KRAS G12D/A*11:01-expressing pancreatic cancer cell lines known to be recognized by the KRAS G12D/A*11:01-restricted TCR (Fig. 6a) [14]. For the five tumors with HLA-A11 expression (the 4432 organoid, Panc-1 and the three tumor lines stably transduced with HLA-A11) where IFN-g release data could be obtained, the level of KRAS G12D expression correlated with their degree of recognition by this TCR, quantified by amount of IFN-γ secretion (*R*^2^ = 0.9916, *p* < 0.0001) (Fig. 6b). Although this result is based on very few samples, in view of the range of expression seen, variations in endogenous levels of mutated KRAS may be a significant parameter in the immune recognition of tumors.

### PDTO can be used to screen TIL for reactivity and isolate individualized TCRs

**Fig. 7:**
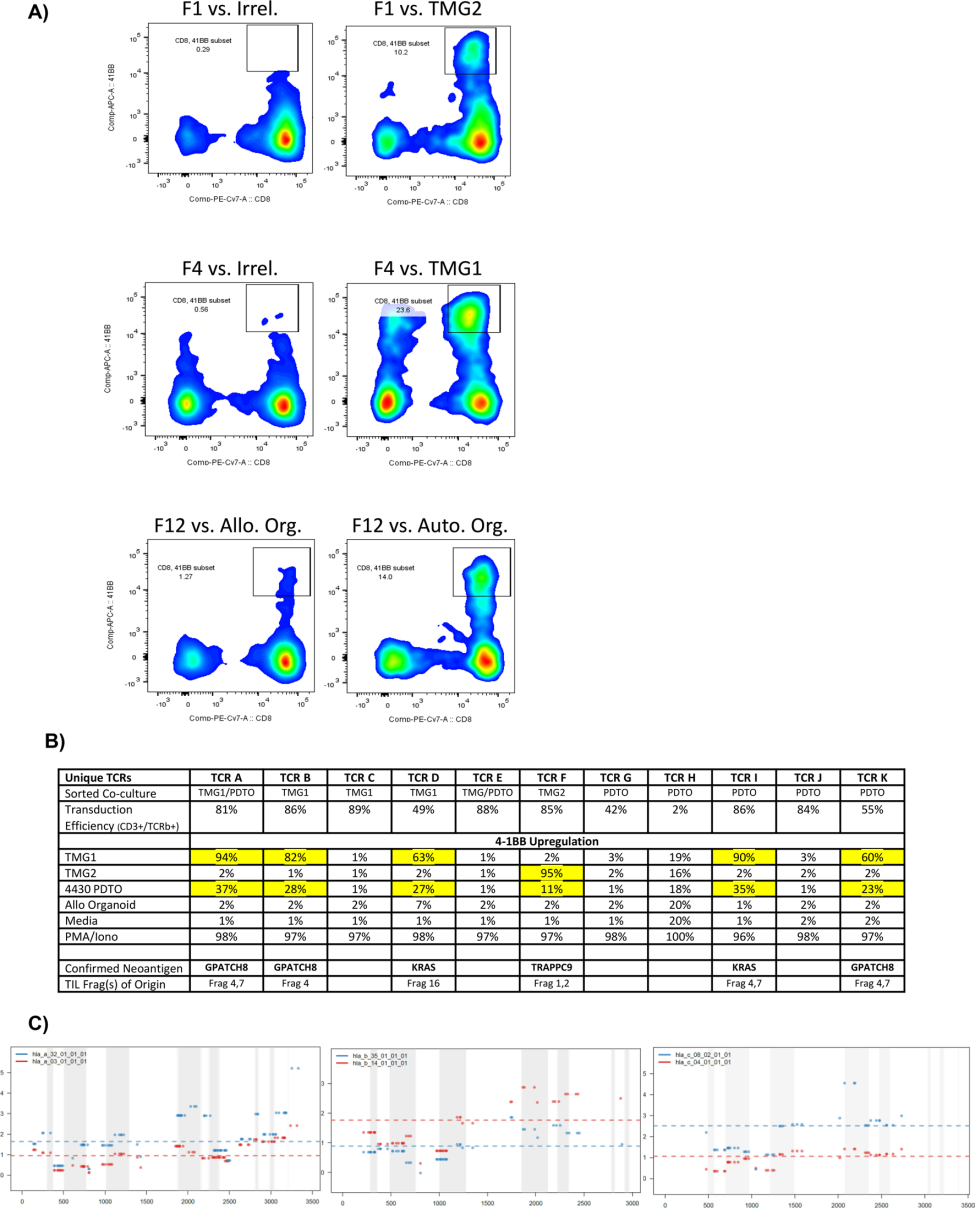
TIL fragments from the patient in Fig 2 were co-cultured with the autologous PDTO as well as with dendritic cells electroporated with TMGs. **a** examples of fragment cultures co-cultured with DC expressing irrelevant TMG, TMG2, TMG 1 or with autologous and allogeneic PDTOs. These co-cultures were sorted for T-cells upregulating 4-1BB to enrich for reactivity and these cells underwent TCR sequencing to identify high-frequency individualized TCRs (iTCRs). **b** Eleven candidate iTCRs were synthesized and retrovirally transduced onto PBL and tested against organoid, TMG 1, and TMG 2. Flow cytometry was performed, measuring percent of CD3+ T-cells upregulating 41BB. TCRs recognizing target cells are highlighted in yellow. Antigen identities that were further confirmed by specific recognition of mutated vs wild type peptide are listed as Confirmed Neoantigens. **c** HLA-LOH analysis was performed, demonstrating that heterozygosity of MHC-Class I A, B and C alleles was maintained in the PDTO

Discovering neoantigen reactive TCRs in TIL using minigenes or mutated peptides presented by autologous APC is a slow and laborious process. In addition, clinical success has already been achieved by directly using TIL in selected patients. We therefore sought to determine if PDTO could be used to directly screen TIL grown from fresh tumor fragments, possibly even bypassing the need for WES of tumors. The previously described PDTO (4430) was co-cultured with autologous TIL cultures from 24 separate tumor fragments (cultures F1–F24) grown as previously described [37]. A parallel analysis using the standard co-culture with autologous dendritic cells electroporated with TMGs was also performed as shown in Fig. 2. CD8 + reactivities to organoid were found in TIL fragment cultures F1, F2, F4, F7, F12, F16 and F23 among others. APC electroporated with TMGs 1 and 2 also stimulated TIL fragment cultures. Sorting these co-cultures for T-cells that upregulated 4-1BB (Fig. 7a) and TCR sequencing eventually yielded 11 dominant and clonally unique TCRs. These were synthesized and individually retrovirally transduced onto donor PBL and retested and their neoantigen targets identified (Fig. 7b). Five of these candidate TCRs were either not neoantigen-specific or their reactivity could not be confirmed. Six of these TCRs were confirmed to recognize the autologous PDTO, targeting three clonal neoantigens (also validated by recognition of the mutated peptide but not wild type). All of these TCRs could be confirmed to also recognize TMG1 or TMG2, validating the accuracy of organoid reactivity. Interestingly, one KRAS G12D-reactive TCR from T-cells retrieved from an organoid co-culture (TCR I) proved to be a different clonotype from the TCR with the same reactivity isolated from the co-culture with TMG1 (TCR D). All six of these TCRs were restricted by MHC Class I, which organoid reactivity predicted was intact; this was confirmed by LOHHLA in the organoid (Fig. 7c).

## Discussion

The basis of adoptive cell therapy is rooted in the fundamental understanding that immune recognition is predicated on specific binding of TCRs to MHC-peptide complexes on the surfaces of tumor cells. For many years, the obstacle has been to find safe immunogenic target antigens on tumors to attack. The discovery that neoantigens are principal drivers of the T-cell response in most successful immunotherapies has addressed the antigen problem for many tumors. In the ideal situation, cancer cells would express sufficient tumor neoantigens that subsequently undergo intracellular processing, get loaded onto MHC molecules, and then are presented on the cell surface for detection by T-cells. This is the first step in clinical tumor rejection, but due to the private nature of tumor neoantigens and the repertoire against them, it has been difficult to study this step in a patient-specific manner. Most of the common epithelial cancers do not grow in two-dimensional cell culture and until recently, there was a paucity of T-cells of defined reactivity against neoantigens. Methods for identifying neoantigen-reactive T-cells from patients with cancer and cloning their TCRs for retroviral re-expression in PBL have been recently described, solving the latter problem [11, 36]. We investigated whether three-dimensional organoid technology could provide a source of autologous tumors with a high degree of fidelity to the tumor of origin and allow immune recognition studies. Failure to achieve tumor recognition and clinical response may be due to both T-cell-specific and tumor-specific causes. We sought to determine whether in vitro use of organoids and cloned neoantigen-reactive T-cells could be used to study failures of tumor recognition caused by tumor-specific defects. If so, they could also be a valuable tool for selecting T-cells for therapy that have retained the ability to recognize autologous tumor expressing physiological levels of antigen.

Consistent with previous publications [20–23], our experience has demonstrated that PDTO can be reliably generated from colorectal, breast, and pancreatic cancers and for most patients, this occurs in a time frame compatible with expanding TIL for evaluation. These lines also displayed a high degree of tumor purity based on WES estimates. Perhaps this is due to their metastatic origin—where the tumor histology differs from the normal tissue at the site of metastasis—and the histology-specific media they are grown in, which promotes tumor organoid growth without supporting normal tissue growth. This feature is important in resolving uncertainties in WES data from fresh tumors with a low tumor cell fraction. For instance, it has been difficult to assess expression of individual MHC alleles by tumors when they are contaminated with large amounts of normal tissues. In our samples, organoid sequencing data was able to resolve this. Also in concordance with other groups, we found that PDTO showed genomic fidelity with the fresh tumor samples from which they were derived [26]. The availability of multiple independent WES from different samples within lesions and between different lesions within a patient showed that the critical clonal mutations of a patient’s tumor were particularly well-conserved between fresh tumor and PTDO. Discrepancies were largely confined to minor subclonal mutations and could be due to either genetic drift during PTDO establishment or random sampling of pre-existing variations with the cancer. Because the focus of T-cell immunotargeting should be the dominant, clonal antigens, these subclonal variations are of lesser significance.

Given these findings that PDTO accurately reflect the mutanome of their source tumor, we evaluated their recognition by T-cells by functional in vitro testing. Using cloned TCRs of defined specificity and MHC restriction, we found a surprising proportion of PDTO harboring the target neoantigen were not recognized. A well-known cause such as β-2 microglobulin loss [37] is likely to be rare as it requires losing both copies of this gene. Loss of the single gene encoding the heavy chain of the MHC-restriction element is much more likely and has been increasingly recognized. Most of these studies use loss of heterozygosity (LOH) in the MHC locus as a surrogate for this event, without defining T-cell reactivities or the precise MHC allele involved. With this metric, LOH at the HLA locus occurred in 11–50% of patients with breast, colon, brain, lung, and pancreatic cancers [38–40]. Other studies show a similar overall rate of HLA LOH, but a much higher frequency of losing a relevant HLA locus in patients with mutations of known immunogenicity [39]. Detecting LOH by sequencing fresh tumor is unreliable when the tumor cell fraction is low and is exacerbated by the polymorphism in the HLA locus and consequent poor sequencing coverage. The availability of PDTO also allows functional validation of in silico findings. We also found that other mechanisms beside HLA loss were responsible for poor recognition of PDTO. When both the mutated neoantigen and the presenting MHC allele were genetically present, some T-cells expressing a TCR of proven specificity for this peptide-MHC complex still did not recognize the PDTO. In many cases, this lack of recognition could be rectified by augmenting the neoantigen epitope by exogenous pulsing of the synthetic minimal determinant, demonstrating the presence of functional MHC molecules. This implies that the native levels of processed peptide-MHC complex are simply inadequate. This may be because the neoantigen target (in this case mutated KRAS) is not required in large amounts to be oncogenic, or that another defect in the antigen processing pathway is present. Organoids may be valuable in defining the landscape of defects preventing successful T-cell therapies or in assessing the adequacy of a TCR selected for the engineering of PBL for transfer.

Other groups have attempted to generate neoantigen-specific T-cell populations by in vitro stimulation of peripheral blood lymphocytes with PDTO lines [41]. This can be slow, laborious, and low-yield, perhaps due to the very low frequency of neoantigen reactive T-cells in the peripheral blood. The use of TIL which are greatly enriched for neoantigen reactivity and rapidly cloning the TCRs with the desired reactivity based on PDTO recognition not only found TCRs but discovered new TCR clonotypes not found by screening with TMG or synthetic peptides. This standard screening also uses normal autologous APC to present candidate neoantigens and would fail to detect defects in antigen processing and presentation present specifically in tumor. Ultimately, the use of PDTO in the primary screening of TIL for administration may replace TMG and peptide screening with the added benefit of editing out antigens which have acquired defects in their processing or presentation.

There are some limitations in this study and with PDTO in general. First, the sample size presented here is small and a much larger survey of PDTO recognition is needed. Nevertheless, this limited sample has already revealed information about patient-specific tumor immune evasion. In addition, when PDTO are generated from a single tumor sample, they may be subject to sampling error and not reflect the full heterogeneity of a patient’s cancer. Fortunately, it appears that the most important clonal mutations are least susceptible to sampling error. Still, the intra-patient heterogeneity of immune evasion mechanisms is unknown and may affect the utility of PDTO. Our recognition studies also lack rigorous negative controls. Finding HLA matched organoids is not practical and establishing matched normal tissue organoids greatly increases the task. Furthermore, it is not clear that a normal lung organoid is the proper control for a colon cancer organoid derived from a lung metastasis. Therefore, many of the tentative finding from organoid studies need correlation with sequencing and other information sources. Ultimately, a study determining whether PDTO recognition correlates with or predicts response to adoptive T-cell transfer will be needed to evaluate their clinical utility.

Mechanistic studies of the immune response to the private neoantigens that drive tumor rejection in patients has been hampered by not having the autologous tumor as a laboratory reagent. This has been particularly true of the common epithelial cancers as opposed to melanoma where much progress was made partially due to its ability to grow in culture. Three-dimensional organoid culture techniques may finally address this need for the cancers which most commonly afflict patients. Combining this capability with the new availability of cloned, defined neoantigen-reactive T-cells from cancer patients may finally elucidate the factors that are blocking successful T-cell therapies.

### Supplementary Information

Below is the link to the electronic supplementary material.

Supplementary file1 (PPTX 602 KB)

## Data Availability

The data generated in this study are available within the article and its supplementary data files.
