# Supplementary material for: Using patient-derived tumor organoids from common epithelial cancers to analyze personalized T-cell responses to neoantigens
Source: Cancer Immunol Immunother. 2023 Jun 27;72(10):3149–62. doi: 10.1007/s00262-023-03476-6 (PMC10491521; doi:10.1007/s00262-023-03476-6)
Supplement: Supplementary file 1 — Supplementary file1 (PPTX 602 KB) [file 262_2023_3476_MOESM1_ESM.pptx]

## Slide 1
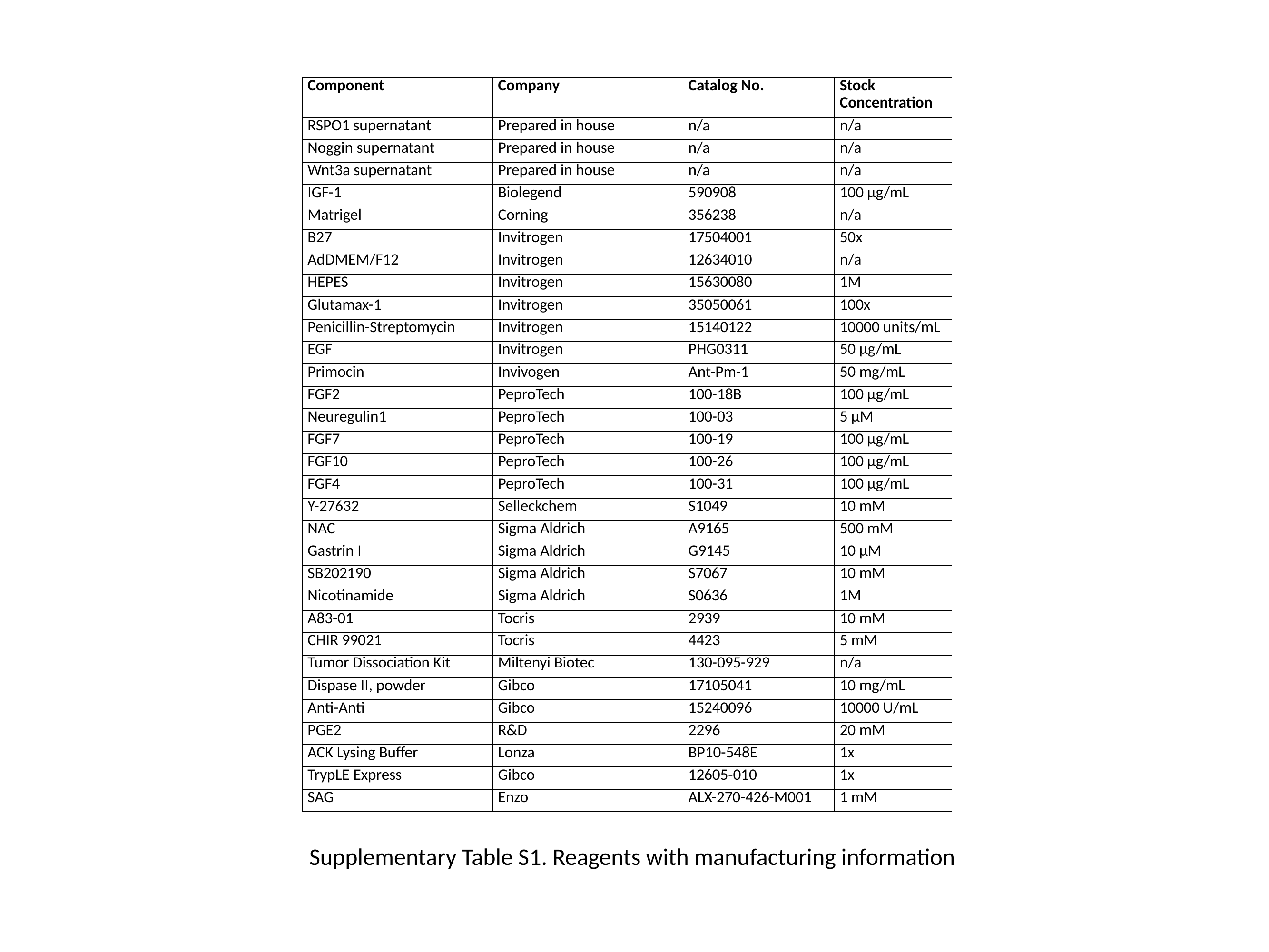

| Component | Company | Catalog No. | Stock Concentration |
| --- | --- | --- | --- |
| RSPO1 supernatant | Prepared in house | n/a | n/a |
| Noggin supernatant | Prepared in house | n/a | n/a |
| Wnt3a supernatant | Prepared in house | n/a | n/a |
| IGF-1 | Biolegend | 590908 | 100 μg/mL |
| Matrigel | Corning | 356238 | n/a |
| B27 | Invitrogen | 17504001 | 50x |
| AdDMEM/F12 | Invitrogen | 12634010 | n/a |
| HEPES | Invitrogen | 15630080 | 1M |
| Glutamax-1 | Invitrogen | 35050061 | 100x |
| Penicillin-Streptomycin | Invitrogen | 15140122 | 10000 units/mL |
| EGF | Invitrogen | PHG0311 | 50 μg/mL |
| Primocin | Invivogen | Ant-Pm-1 | 50 mg/mL |
| FGF2 | PeproTech | 100-18B | 100 μg/mL |
| Neuregulin1 | PeproTech | 100-03 | 5 μM |
| FGF7 | PeproTech | 100-19 | 100 μg/mL |
| FGF10 | PeproTech | 100-26 | 100 μg/mL |
| FGF4 | PeproTech | 100-31 | 100 μg/mL |
| Y-27632 | Selleckchem | S1049 | 10 mM |
| NAC | Sigma Aldrich | A9165 | 500 mM |
| Gastrin I | Sigma Aldrich | G9145 | 10 μM |
| SB202190 | Sigma Aldrich | S7067 | 10 mM |
| Nicotinamide | Sigma Aldrich | S0636 | 1M |
| A83-01 | Tocris | 2939 | 10 mM |
| CHIR 99021 | Tocris | 4423 | 5 mM |
| Tumor Dissociation Kit | Miltenyi Biotec | 130-095-929 | n/a |
| Dispase II, powder | Gibco | 17105041 | 10 mg/mL |
| Anti-Anti | Gibco | 15240096 | 10000 U/mL |
| PGE2 | R&D | 2296 | 20 mM |
| ACK Lysing Buffer | Lonza | BP10-548E | 1x |
| TrypLE Express | Gibco | 12605-010 | 1x |
| SAG | Enzo | ALX-270-426-M001 | 1 mM |
Supplementary Table S1. Reagents with manufacturing information

## Slide 2
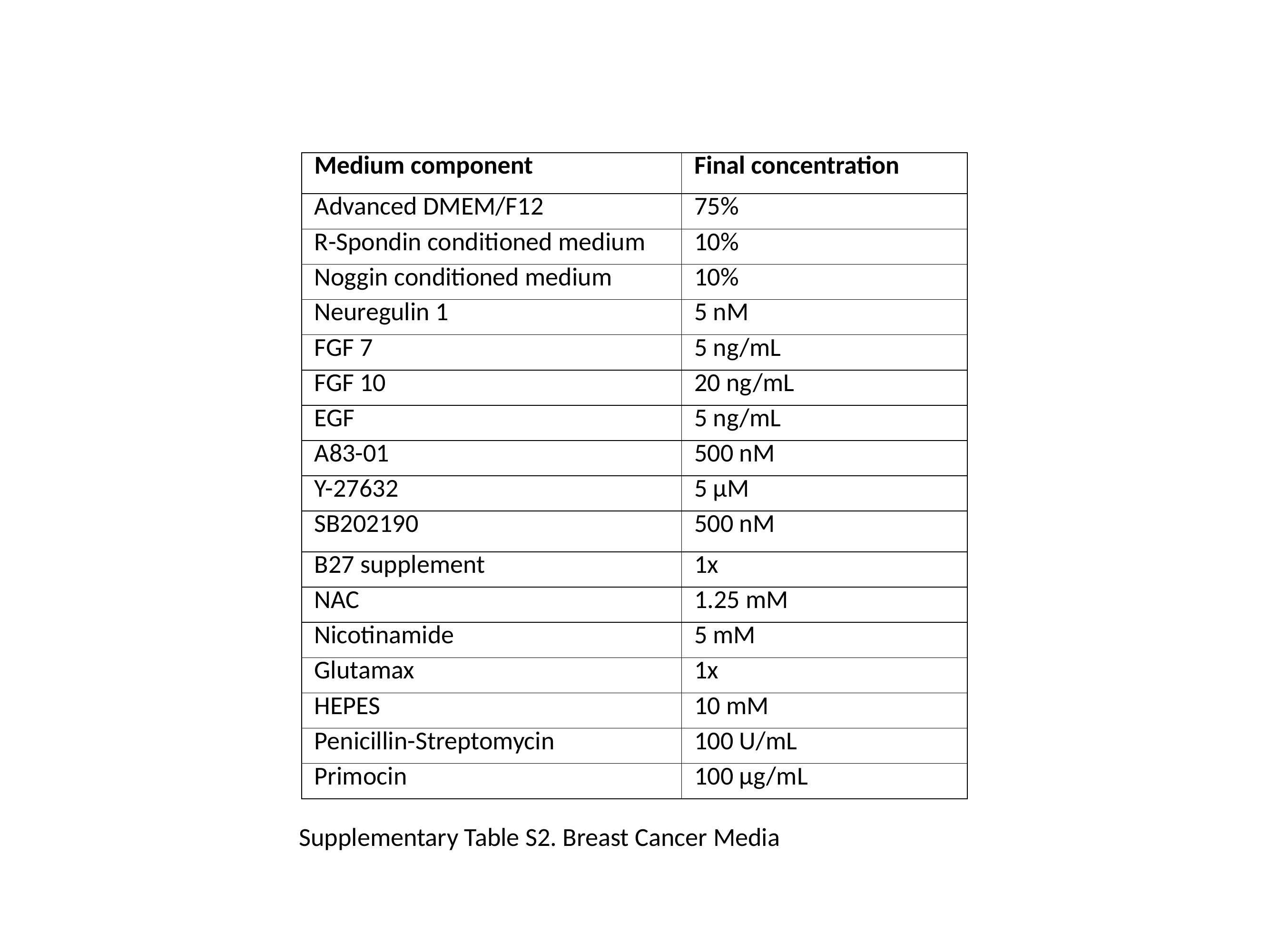

| Medium component | Final concentration |
| --- | --- |
| Advanced DMEM/F12 | 75% |
| R-Spondin conditioned medium | 10% |
| Noggin conditioned medium | 10% |
| Neuregulin 1 | 5 nM |
| FGF 7 | 5 ng/mL |
| FGF 10 | 20 ng/mL |
| EGF | 5 ng/mL |
| A83-01 | 500 nM |
| Y-27632 | 5 μM |
| SB202190 | 500 nM |
| B27 supplement | 1x |
| NAC | 1.25 mM |
| Nicotinamide | 5 mM |
| Glutamax | 1x |
| HEPES | 10 mM |
| Penicillin-Streptomycin | 100 U/mL |
| Primocin | 100 μg/mL |
Supplementary Table S2. Breast Cancer Media

## Slide 3
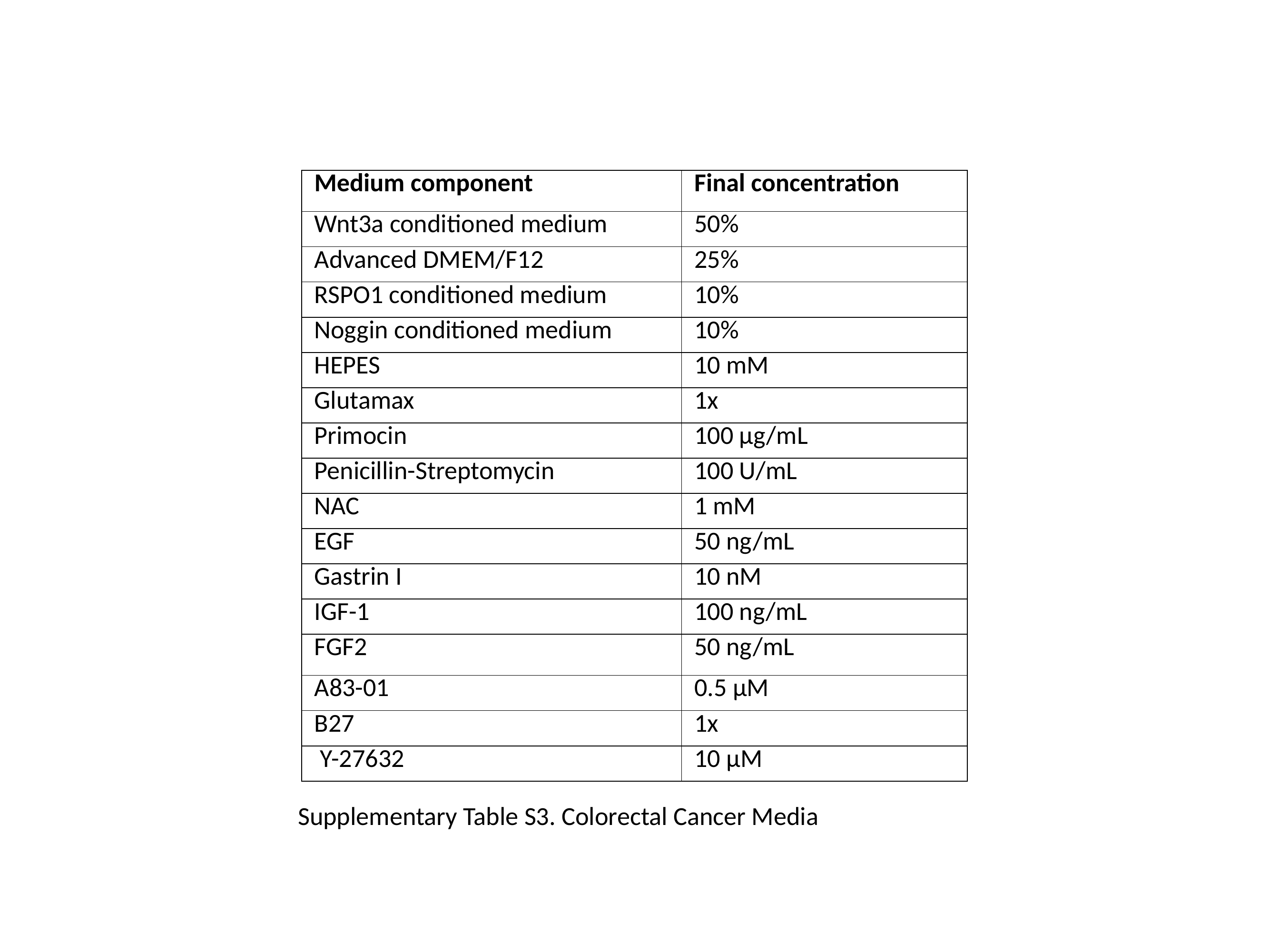

| Medium component | Final concentration |
| --- | --- |
| Wnt3a conditioned medium | 50% |
| Advanced DMEM/F12 | 25% |
| RSPO1 conditioned medium | 10% |
| Noggin conditioned medium | 10% |
| HEPES | 10 mM |
| Glutamax | 1x |
| Primocin | 100 μg/mL |
| Penicillin-Streptomycin | 100 U/mL |
| NAC | 1 mM |
| EGF | 50 ng/mL |
| Gastrin I | 10 nM |
| IGF-1 | 100 ng/mL |
| FGF2 | 50 ng/mL |
| A83-01 | 0.5 μM |
| B27 | 1x |
| Y-27632 | 10 μM |
Supplementary Table S3. Colorectal Cancer Media

## Slide 4
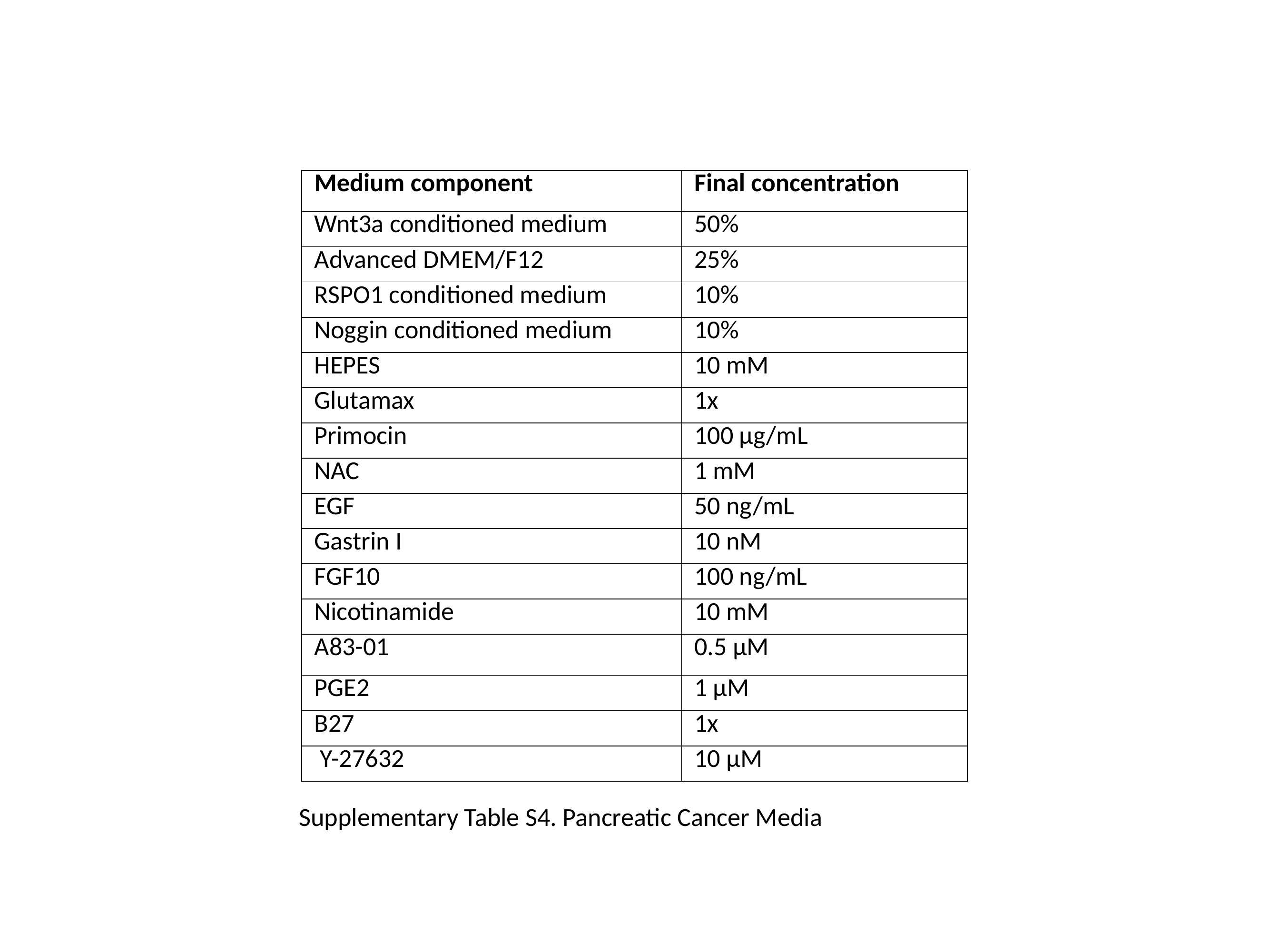

| Medium component | Final concentration |
| --- | --- |
| Wnt3a conditioned medium | 50% |
| Advanced DMEM/F12 | 25% |
| RSPO1 conditioned medium | 10% |
| Noggin conditioned medium | 10% |
| HEPES | 10 mM |
| Glutamax | 1x |
| Primocin | 100 μg/mL |
| NAC | 1 mM |
| EGF | 50 ng/mL |
| Gastrin I | 10 nM |
| FGF10 | 100 ng/mL |
| Nicotinamide | 10 mM |
| A83-01 | 0.5 μM |
| PGE2 | 1 μM |
| B27 | 1x |
| Y-27632 | 10 μM |
Supplementary Table S4. Pancreatic Cancer Media

## Slide 5
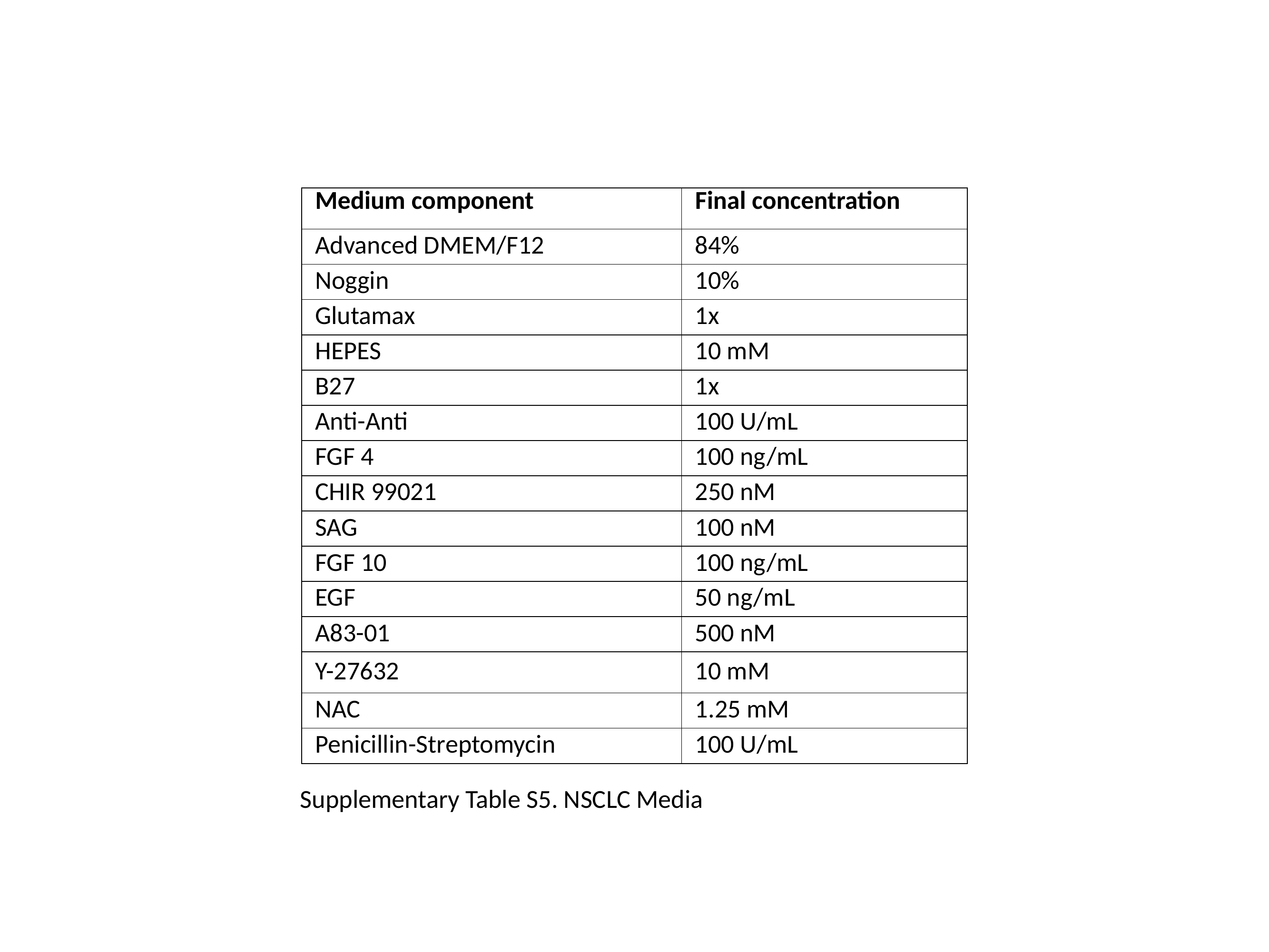

| Medium component | Final concentration |
| --- | --- |
| Advanced DMEM/F12 | 84% |
| Noggin | 10% |
| Glutamax | 1x |
| HEPES | 10 mM |
| B27 | 1x |
| Anti-Anti | 100 U/mL |
| FGF 4 | 100 ng/mL |
| CHIR 99021 | 250 nM |
| SAG | 100 nM |
| FGF 10 | 100 ng/mL |
| EGF | 50 ng/mL |
| A83-01 | 500 nM |
| Y-27632 | 10 mM |
| NAC | 1.25 mM |
| Penicillin-Streptomycin | 100 U/mL |
Supplementary Table S5. NSCLC Media

## Slide 6
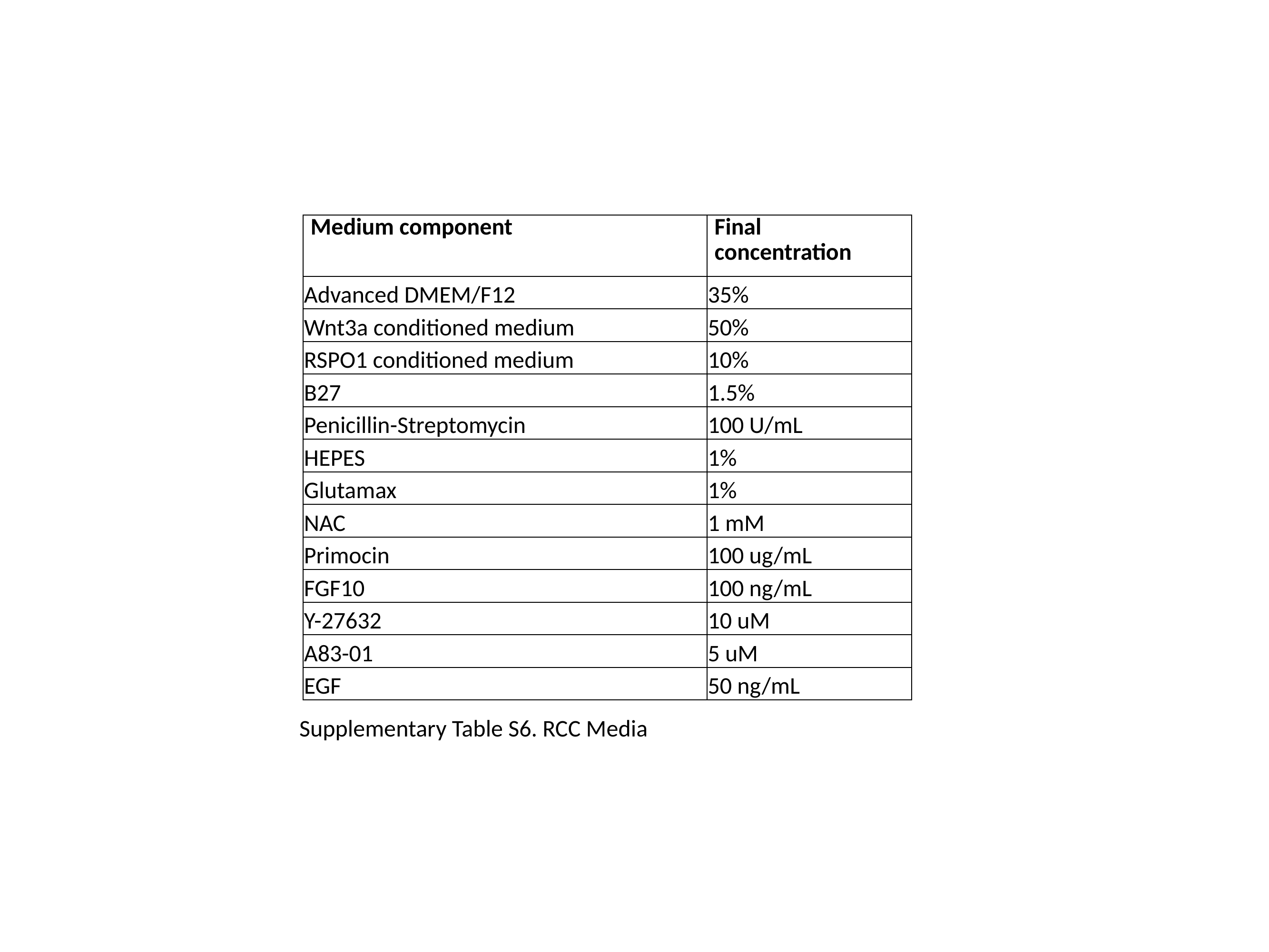

| Medium component | Final concentration |
| --- | --- |
| Advanced DMEM/F12 | 35% |
| Wnt3a conditioned medium | 50% |
| RSPO1 conditioned medium | 10% |
| B27 | 1.5% |
| Penicillin-Streptomycin | 100 U/mL |
| HEPES | 1% |
| Glutamax | 1% |
| NAC | 1 mM |
| Primocin | 100 ug/mL |
| FGF10 | 100 ng/mL |
| Y-27632 | 10 uM |
| A83-01 | 5 uM |
| EGF | 50 ng/mL |
Supplementary Table S6. RCC Media

## Slide 7
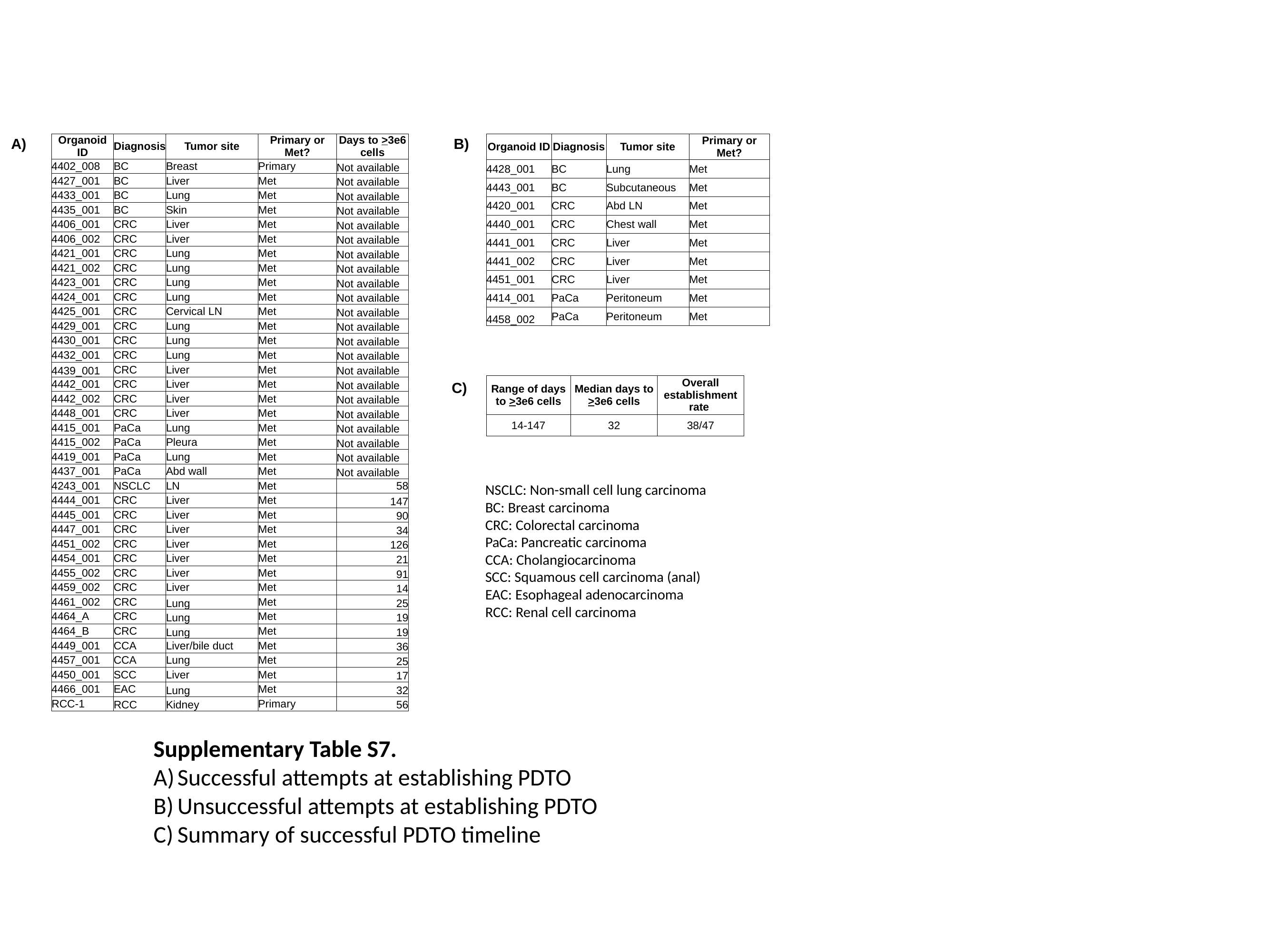

A)
B)
| Organoid ID | Diagnosis | Tumor site | Primary or Met? | Days to >3e6 cells |
| --- | --- | --- | --- | --- |
| 4402\_008 | BC | Breast | Primary | Not available |
| 4427\_001 | BC | Liver | Met | Not available |
| 4433\_001 | BC | Lung | Met | Not available |
| 4435\_001 | BC | Skin | Met | Not available |
| 4406\_001 | CRC | Liver | Met | Not available |
| 4406\_002 | CRC | Liver | Met | Not available |
| 4421\_001 | CRC | Lung | Met | Not available |
| 4421\_002 | CRC | Lung | Met | Not available |
| 4423\_001 | CRC | Lung | Met | Not available |
| 4424\_001 | CRC | Lung | Met | Not available |
| 4425\_001 | CRC | Cervical LN | Met | Not available |
| 4429\_001 | CRC | Lung | Met | Not available |
| 4430\_001 | CRC | Lung | Met | Not available |
| 4432\_001 | CRC | Lung | Met | Not available |
| 4439\_001 | CRC | Liver | Met | Not available |
| 4442\_001 | CRC | Liver | Met | Not available |
| 4442\_002 | CRC | Liver | Met | Not available |
| 4448\_001 | CRC | Liver | Met | Not available |
| 4415\_001 | PaCa | Lung | Met | Not available |
| 4415\_002 | PaCa | Pleura | Met | Not available |
| 4419\_001 | PaCa | Lung | Met | Not available |
| 4437\_001 | PaCa | Abd wall | Met | Not available |
| 4243\_001 | NSCLC | LN | Met | 58 |
| 4444\_001 | CRC | Liver | Met | 147 |
| 4445\_001 | CRC | Liver | Met | 90 |
| 4447\_001 | CRC | Liver | Met | 34 |
| 4451\_002 | CRC | Liver | Met | 126 |
| 4454\_001 | CRC | Liver | Met | 21 |
| 4455\_002 | CRC | Liver | Met | 91 |
| 4459\_002 | CRC | Liver | Met | 14 |
| 4461\_002 | CRC | Lung | Met | 25 |
| 4464\_A | CRC | Lung | Met | 19 |
| 4464\_B | CRC | Lung | Met | 19 |
| 4449\_001 | CCA | Liver/bile duct | Met | 36 |
| 4457\_001 | CCA | Lung | Met | 25 |
| 4450\_001 | SCC | Liver | Met | 17 |
| 4466\_001 | EAC | Lung | Met | 32 |
| RCC-1 | RCC | Kidney | Primary | 56 |
| Organoid ID | Diagnosis | Tumor site | Primary or Met? |
| --- | --- | --- | --- |
| 4428\_001 | BC | Lung | Met |
| 4443\_001 | BC | Subcutaneous | Met |
| 4420\_001 | CRC | Abd LN | Met |
| 4440\_001 | CRC | Chest wall | Met |
| 4441\_001 | CRC | Liver | Met |
| 4441\_002 | CRC | Liver | Met |
| 4451\_001 | CRC | Liver | Met |
| 4414\_001 | PaCa | Peritoneum | Met |
| 4458\_002 | PaCa | Peritoneum | Met |
C)
| Range of days to >3e6 cells | Median days to >3e6 cells | Overall establishment rate |
| --- | --- | --- |
| 14-147 | 32 | 38/47 |
NSCLC: Non-small cell lung carcinoma
BC: Breast carcinoma
CRC: Colorectal carcinoma
PaCa: Pancreatic carcinoma
CCA: Cholangiocarcinoma
SCC: Squamous cell carcinoma (anal)
EAC: Esophageal adenocarcinoma
RCC: Renal cell carcinoma
Supplementary Table S7.
Successful attempts at establishing PDTO
Unsuccessful attempts at establishing PDTO
Summary of successful PDTO timeline

## Slide 8
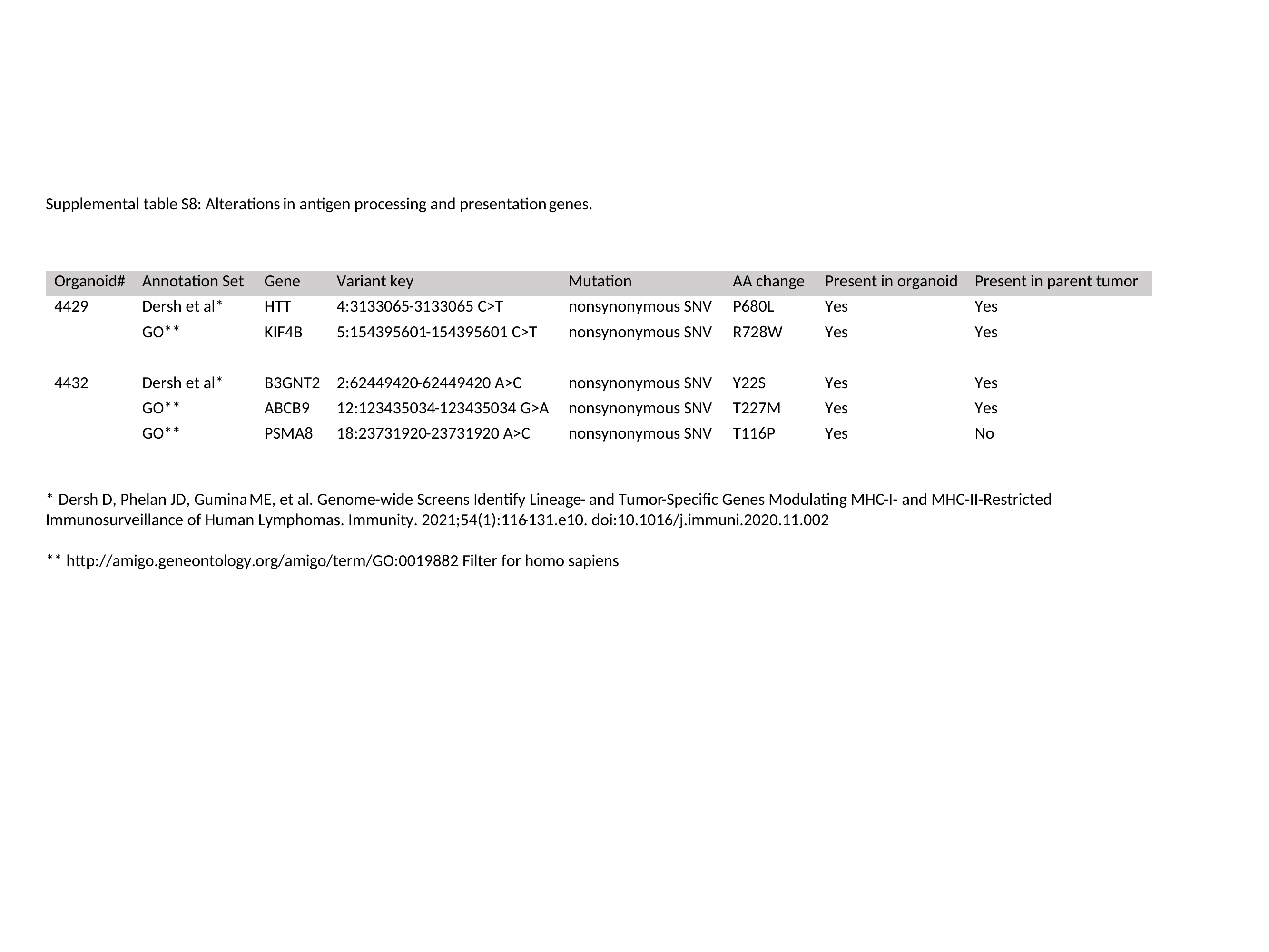

## Slide 9
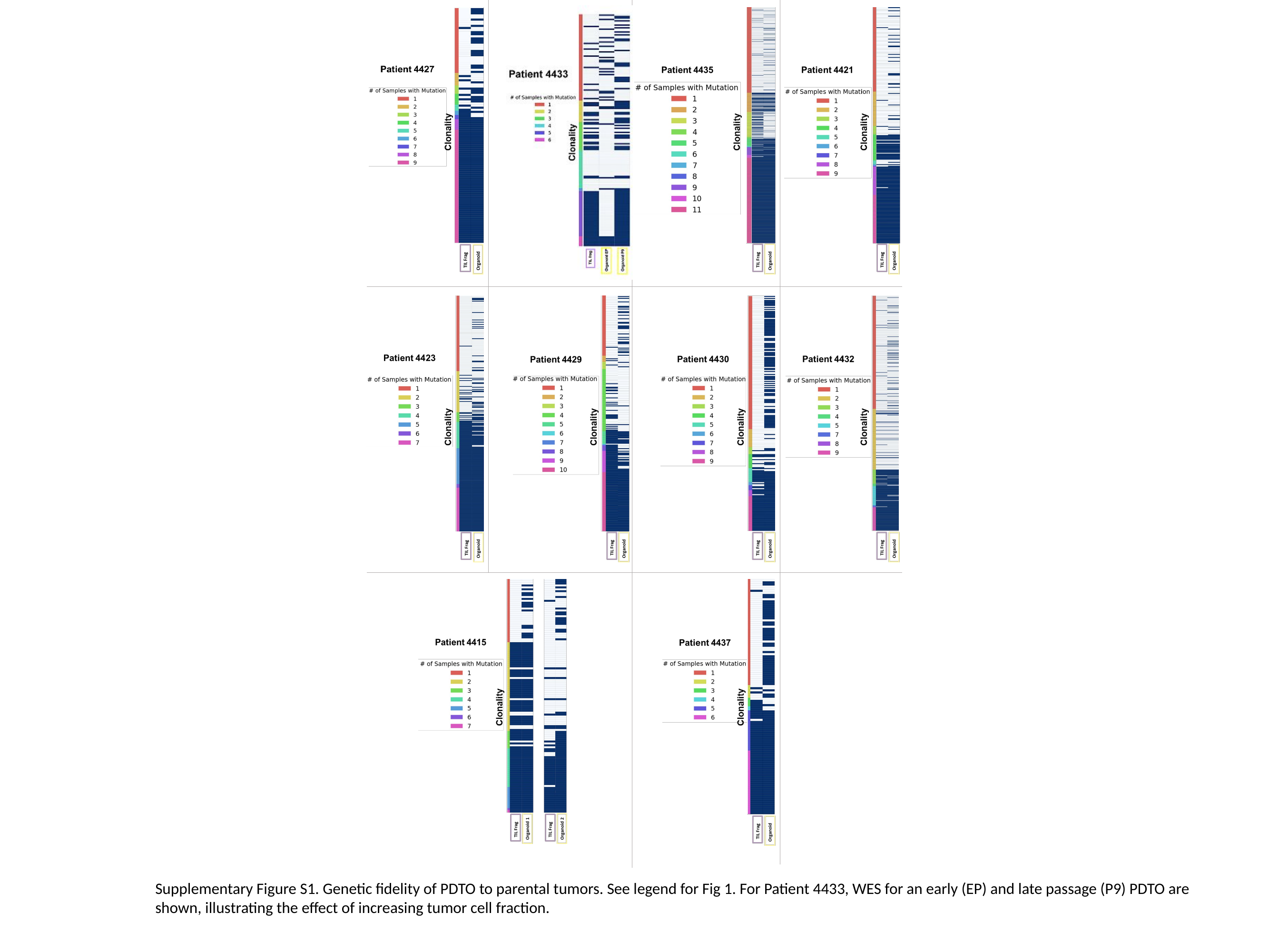

Supplementary Figure S1. Genetic fidelity of PDTO to parental tumors. See legend for Fig 1. For Patient 4433, WES for an early (EP) and late passage (P9) PDTO are shown, illustrating the effect of increasing tumor cell fraction.
